# *In vivo* physiological recording from the lateral line of juvenile zebrafish

**DOI:** 10.1113/JP271794

**Published:** 2016-06-09

**Authors:** Jennifer Olt, Claire E. Allen, Walter Marcotti

**Affiliations:** ^1^ Department of Biomedical ScienceUniversity of SheffieldSheffieldS10 2TNUK

## Abstract

**Key points:**

- Zebrafish provide a unique opportunity to investigate *in vivo* sensory transduction in mature hair cells.
- We have developed a method for studying the biophysical properties of mature hair cells from the lateral line of juvenile zebrafish.
- The method involves application of the anaesthetic benzocaine and intubation to maintain ventilation and oxygenation through the gills.
- The same approach could be used for *in vivo* functional studies in other sensory and non‐sensory systems from juvenile and adult zebrafish.

**Abstract:**

Hair cells are sensory receptors responsible for transducing auditory and vestibular information into electrical signals, which are then transmitted with remarkable precision to afferent neurons. The zebrafish lateral line is emerging as an excellent *in vivo* model for genetic and physiological analysis of hair cells and neurons. However, research has been limited to larval stages because zebrafish become protected from the time of independent feeding under European law (from 5.2 days post‐fertilization (dpf) at 28.5°C). In larval zebrafish, the functional properties of most of hair cells, as well as those of other excitable cells, are still immature. We have developed an experimental protocol to record electrophysiological properties from hair cells of the lateral line in juvenile zebrafish. We found that the anaesthetic benzocaine at 50 mg l^−1^ was an effective and safe anaesthetic to use on juvenile zebrafish. Concentrations up to 300 mg l^−1^ did not affect the electrical properties or synaptic vesicle release of juvenile hair cells, unlike the commonly used anaesthetic MS‐222, which reduces the size of basolateral membrane K^+^ currents. Additionally, we implemented a method to maintain gill movement, and as such respiration and blood oxygenation, via the intubation of > 21 dpf zebrafish. The combination of benzocaine and intubation provides an experimental platform to investigate the physiology of mature hair cells from live zebrafish. More generally, this method would allow functional studies involving live imaging and electrophysiology from juvenile and adult zebrafish.

## Key points

- Zebrafish provide a unique opportunity to investigate *in vivo* sensory transduction in mature hair cells.
- We have developed a method for studying the biophysical properties of mature hair cells from the lateral line of juvenile zebrafish.
- The method involves application of the anaesthetic benzocaine and intubation to maintain ventilation and oxygenation through the gills.
- The same approach could be used for *in vivo* functional studies in other sensory and non‐sensory systems from juvenile and adult zebrafish.

**Abbreviations**
dpf: days post‐fertilization
MET: mechanoelectrical transducer
OHC: outer hair cell
PLLg: posterior lateral line ganglion
wpf: weeks post fertilization

## Introduction

The zebrafish is becoming an increasingly popular model not only to study the genetic basis of hearing and deafness (Grunwald & Eisen 2002; Nicolson, 2005) but also to investigate the molecular mechanisms controlling the normal development and function of sensory hair cells (Kindt *et al*. 2012; Sheets *et al*. 2012; Maeda *et al*. 2014; Olt *et al*. 2014). However, there are at least three major limitations with using the zebrafish lateral line as an *in vivo* model. First of all, the commonly used anaesthetic MS‐222 (tricaine methanesulfonate) is an effective blocker of K^+^ currents expressed in hair cells (Olt *et al*. 2014). Secondly, zebrafish become protected in the European Union from the day of independent feeding at larval stage (Directive 2010/63/EU), which at 28.5°C holding temperature corresponds to 5.2 days post‐fertilization (dpf). Therefore, *in vivo* physiological experiments are normally performed at embryonic or larval stages when the majority of hair cells within a neuromast have an immature synaptic activity and basolateral current profile (Olt *et al*. 2014). Finally, oxygenation in > 21 dpf zebrafish is entirely dependent on gill function, which prevents *in vivo* experiments at older ages. This limits the use of zebrafish for studies on mature physiological characteristics, not only in the lateral line (Liao & Haehnel, 2012; Olt *et al*. 2014) but also in other systems such as the brain (Vargas *et al*. 2011).

Mechanosensory hair cells are specialized to detect stimuli, such as sound, head movement and water flow, with precision and to convey these signals to afferent neurons via specialized ribbon synapses (Corns *et al*. 2014). Our understanding of the physiological mechanisms underlying the development and function of mammalian hair cells comes from *in vitro* cochlear preparations because *in vivo* experiments are not currently possible in altricial rodents. Similar to hair cells in the auditory and vestibular systems of lower vertebrates and mammals (Fettiplace & Hackney, 2006), hair cells within the neuromasts of the lateral line transduce motion of their stereociliary hair bundles into an electrical signal that is transmitted to the afferent fibres via ribbon synapses (Nicolson, 2005, 2015). The zebrafish uses the lateral line to signal the perception of hydrodynamics around the body, which is used for orientation and coordination of motor behaviour (Bleckmann & Zelick, 2009). Here we developed a method that allows *in vivo* electrophysiological recordings from the lateral line of > 21 dpf zebrafish using the anaesthetic benzocaine alongside gill oxygenation via a cannula inserted into their mouth. The ability to perform *in vivo* electrophysiological recordings from juvenile zebrafish will provide a tool to gain a better understanding of physiological processes in the lateral line and other sensory systems, thus extending the experimental potential of this animal model to perform basic research and to study human pathologies.

## Methods

### Ethics statement

All experiments were performed in accordance with Home Office regulations under the Animals (Scientific Procedures Act) 1986 and following approval by the University of Sheffield Ethical Review Committee.

### Tissue preparation

Hair cell recordings were performed from the primary neuromasts (L2–L4) originating from the first primordium (primI) (Pujol‐Martí & López‐Schier, 2013). Afferent neuron recordings were obtained from the cell bodies of the posterior lateral line ganglion (PLLg). The different developmental stages of the zebrafish (*Danio rerio*) were classified as previously described (Parichy *et al*. 2009; Olt *et al*. 2014): larval from 3 days post‐fertilization (3 dpf) to ∼2 weeks post‐fertilization (2 wpf); juvenile or young‐adult from ∼2 wpf to when fish become sexually mature (3–6 months); adult from 6 months onwards. Anaesthetized zebrafish were transferred to a microscope chamber, immobilized onto a thin layer of silicone elastomer (Sylgard 184) using 0.015 mm (larval) or 0.025 mm (juvenile) diameter tungsten wire and continuously perfused with the following extracellular solution (in mm): 135 NaCl, 1.3 CaCl_2_, 5.8 KCl, 0.9 mgCl_2_, 0.7 NaH_2_PO_4_, 5.6 d‐glucose, 10 Hepes‐NaOH. Sodium pyruvate (2 mm), minimal essential medium (MEM) amino acids solution (50×, without l‐Glutamine) and MEM vitamins solution (100×) were added from concentrates (Fisher Scientific, Loughborough, UK). The pH was 7.5. For Ca^2+^ current (*I*_Ca_) recordings, the extracellular solution was as above except for CaCl_2_, which was increased to 2.8 mm to elicit a larger *I*_Ca_ (osmolality was maintained by reducing NaCl to 133 mm). For cell‐attached recordings, the extracellular solution was as previously reported (Trapani & Nicolson, 2010) and contained (in mm): 140 NaCl, 2.0 CaCl_2_, 2.0 KCl, 1.0 mgCl_2_, 10 Hepes‐NaOH (pH 7.8). Access to the basolateral membrane of the lateral line hair cells for patch‐clamp recordings was obtained in a few steps using a ‘cleaning’ glass pipette (borosilicate glass: O.D. 1.5 mm; I.D. 1.17 mm, Harvard Apparatus, Cambourne, UK) with a diameter of 3–4 μm (for more details see Olt *et al*. 2016).

### Anaesthetics

The anaesthetic tricane methanesulfonate (MS‐222, Henry Schein, Inc., Dumfries, UK) and benzocaine (Sigma‐Aldrich, UK) were used in this study. While benzocaine is classed as a non‐hazardous substance (EC regulation No 1272/2008), MS‐222 is considered to be hazardous to humans with possible risk for skin, eye and respiratory irritation. Therefore, while benzocaine can be prepared using nitrile gloves, MS‐222 must be prepared under a fume hood, wearing protective gloves and respiratory protection. Additional care must be taken when using MS‐222 since it is light sensitive, susceptible to degradation and must be buffered to neutral pH to avoid the irritation of the zebrafish skin. Benzocaine is much less water soluble and as such is normally prepared in 100% ethanol (stock solution: 20 g l^–1^). Like MS‐222, benzocaine is considered to be a safe anaesthetic for fish and amphibians since it does not affect their general health (Neiffer & Stamper, 2009).

The different stages of anaesthesia (Table 1) were assessed based on changes in swimming activity, balance and reaction to external stimuli, which are parameters normally used for fish (Zahl *et al*. 2012). Stages I and II were not assessed because it was too difficult to differentiate between the two stages accurately, which was mainly due to the fact that juvenile zebrafish reached these stages very quickly. At stage III zebrafish stop swimming and lie on their side (loss of equilibrium); the heart beat is normal, respiration is maintained and fish respond to light touch stimuli, such as tapping the tank. At stage IV fish are no longer responsive to light stimuli, while showing normal heart rate and respiration. Stage V was reached when fish no longer reacted to noxious stimuli, such as touching the tail fin with forceps. Heart rate and respiration were still present, although reduced when compared to previous stages. Due to the small size of the juvenile zebrafish, we were not able to quantify with precision the respiration rate (gill movement) and heartbeat, which are normally measured in larger adult fish.

**Table 1:** Time to reach stages of anaesthesia with benzocaine and MS‐222

		Benzocaine		MS‐222
Stage		50 mg l^–1^	100 mg l^–1^	0.017%
I and II	Disorientation and reduced swimming	—	—	—
III	Loss of equilibrium and cessation of swimming activity	3:10 ± 0:40 min (*n* = 6)	1:20 ± 0:10 min (*n* = 7)	1:05 ± 0:20 min (*n* = 7)
IV	No responsive to light stimuli	4:40 ± 0:40 min (*n* = 6)	2:50 ± 0:30 min (*n* = 7)	2:10 ± 0:40 min (*n* = 7)
V	Deep sedation: respiration and heart rate maintained	11:10 ± 0:40 min (*n* = 6)	3:20 ± 0:40 min (*n* = 7)	4:40 ± 1:30 min (*n* = 7)
VI	Death	≥ 56:40 ± 0:40 min (*n* = 3)	35:00 ± 0:00 min (*n* = 3)	43:50 ± 12:30 min (*n* = 7)

Concentrations of the anaesthetic are quoted as normally used in the literature. Juvenile zebrafish length was 15.2 ± 0.9 mm (*n* = 17). The age of the fish tested was between 42 and 47 dpf. Stages I and II were not assessed because it was too difficult to differentiate between the two stages accurately. Values reported represent the time from when the anaesthetic was first perfused onto the zebrafish until the indicated stage of anaesthesia occurred. In 50 mg l^–1^ benzocaine 4 out of 7 fish were culled just after 60 min, and as such were not included in the above average.

For *in vivo* recordings (Fig. 6), juvenile zebrafish were immersed in 50 mg l^–1^ benzocaine until they reached stage V anaesthesia (Table 1). Due to the high magnification of the microscopes used (see next section), it was difficult to monitor the heartbeat and respiration of the zebrafish during the recordings. However, we were able to monitor blood flow, which was easily visualized since zebrafish are still largely transparent at juvenile stages. Recordings were also limited to 40–45 min from the start of the anaesthetic, which was well before death normally occurred (see stage VI anaesthesia in Table 1).

### Electrophysiological recordings

Hair cells in the neuromasts and ganglion cell bodies were viewed through long working‐distance ×60 or ×63 water‐immersion objectives with additional magnification of ×1.5 or ×2.0 positioned before the x15 eyepieces of an upright microscope (Olympus OlBX51WI, UK; Leica DMLMF, Germany) with Nomarski optics. Patch pipettes were made from soda glass capillaries for whole‐cell recordings (3–5 MΩ) and coated with surf wax (Mr Zoggs SexWax; Sexwax Inc., Carpinteria, CA, USA) or borosilicate capillaries for loose‐patch (12–14 MΩ). While whole‐cell recording was used to measure hair cell electrical properties, loose‐patch configuration (seal resistances 80 ± 8 MΩ, *n* = 15) allowed the recording of spontaneous action potential activity from the PLLg (Trapani & Nicolson, 2010, 2011). For K^+^ current recordings the patch pipette filling solution contained (in mm): 131 KCl, 3 mgCl_2_, 1 EGTA‐KOH, 5 Na_2_ATP, 5 Hepes‐KOH, 10 sodium phosphocreatine (pH 7.3). For Ca^2+^ current recordings and capacitance measurements the intracellular solution was (in mm): 85 caesium glutamate, 20 CsCl, 3 mgCl_2_, 1 EGTA‐CsOH, 5 Na_2_ATP, 5 Hepes‐CsOH, 10 sodium phosphocreatine; 0.3 Na_2_GTP, 15 4‐aminopyridine (4‐AP); 20 TEA (pH 7.3). Membrane potentials in whole‐cell voltage clamp were corrected for the voltage drop across the uncompensated residual series resistance and for a liquid junction potential, measured between electrode and bath solutions, of −4 mV for the KCl intracellular solution and −9 mV for caesium glutamate. For cell‐attached recordings, the patch pipette filling solution was the same as that used for bathing the zebrafish (see above). Recordings were made with an Optopatch (Cairn Research, Faversham, UK) or an Axopatch 200B (Molecular Devices, Sunnyvale, CA, USA) amplifier. Data acquisition was performed using pCLAMP software with a Digidata data acquisition board (Molecular Devices). Whole‐cell recordings were sampled at 5 or 100 kHz and low pass filtered at 2.5 or 10 kHz (8‐pole Bessel); loose‐patch recordings were sampled at 5 kHz. All data were stored on computer for off‐line analysis (Origin, OriginLab Corp., Northampton, MA, USA; pCLAMP, Molecular Devices). The Mini Analysis Program (Synaptosoft Inc., Fort Lee, NJ, USA) was used to detect spike events in loose‐patch, to calculate their frequency and to analyse interspike intervals (ISIs). The action potential frequency was calculated as the reciprocal of the mean ISI for each cell and an indication of the spread of ISI values about the mean was obtained by calculating the coefficient of variation (CV), equal to the standard deviation divided by the mean.

Whole‐cell and loose‐patch recordings were primarily performed at room temperature (21–24°C). Calcium current recordings and measurements of exocytosis were conducted at the temperature that zebrafish are normally kept under the husbandry conditions at the University of Sheffield (28.5°C).

### Electrophysiological recordings in mouse outer hair cells

Apical‐coil outer hair cells (OHCs) were studied in acutely dissected cochlea from postnatal day (P)6 mice as previously described (Corns *et al*. 2016). Mechanoelectrical transducer (MET) currents were elicited by stimulating the hair bundles of OHCs using a fluid jet from a pipette driven by a piezoelectric disc (Corns *et al*. 2014). The pipette tip of the fluid jet was positioned near to the bundles to elicit a maximal MET current. Mechanical stimuli were applied as 50 Hz sinusoids (filtered at 0.25 kHz, 8‐pole Bessel) with driving voltages of ±40 V. MET currents were recorded with a patch pipette solution containing (in mM): 106 caesium glutamate, 20 CsCl, 3 mgCl_2_, 1 EGTA‐CsOH, 5 Na_2_ATP, 0.3 Na_2_GTP, 5 Hepes‐CsOH, 10 sodium phosphocreatine (pH 7.3). Membrane potentials were corrected for the liquid junction potential (−11 mV).

### Statistical analysis

Statistical comparisons were made with Student's two‐tailed *t* test or, for multiple comparisons, one‐way ANOVA followed by a Bonferroni *post hoc* test. Mean values are quoted ± standard error of the mean (sem) where *P < *0.05 indicates statistical significance.

### FM1‐43 labelling

The entire zebrafish was briefly (10–15 s) superfused with a solution containing 6 μm FM1‐43 (Gale *et al*. 2001) or 6 μm FM1‐43 together with 50 mg l^–1^ benzocaine and hair cells within each neuromast viewed with an upright microscope equipped with epifluorescence illumination and FITC filters (excitation 488 nm, emission 520 nm) using the optics described above. Images were captured using a CCD camera (SPOT‐JNR). Stock solutions of 3 mm FM1‐43 were prepared in water. In some experiments, FM1‐43 was applied following the exposure (5–6 min) of lateral line hair cells to the Ca^2+^ chelator BAPTA in order to cut both the tip links and the kinocilial links (Assad *et al*. 1991; Goodyear & Richardson, 2003). These experiments were performed at room temperature.

## Results

**Figure 1:**
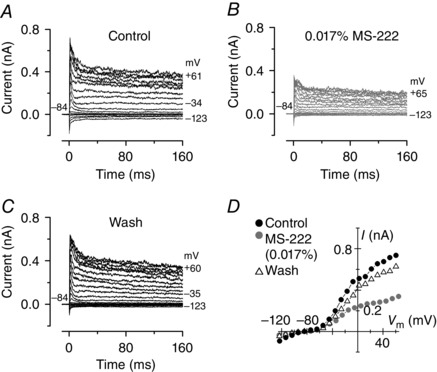
**Effect of MS‐222 on the K^+^ currents recorded from young‐adult lateral line hair cells** *A*–*C*, K^+^ current recordings from hair cell before (*A*), during (*B*) and after (*C*) local superfusion of 0.017% MS‐222. For this recording, the decapitated zebrafish (19 dpf) was maintained in a normal extracellular solution without MS‐222, which was locally superfused onto the patched hair cell. Currents were obtained by applying a series of voltage steps in nominal 10 mV increments from –124 mV, starting from the holding potential at –84 mV. The presence of MS‐222 largely reduced both the peak and steady‐state of the outward K^+^ current (*B*). *D*, peak current–voltage relation obtained from panels *A–C* above.

MS‐222 is one of the most commonly used agents to anaesthetize zebrafish (e.g. Matthews & Varga, 2012; Barbosa *et al*. 2015; Xu *et al*. 2015). However, zebrafish are known to become more sensitive to MS‐222 with development (Rombough, 2007) and it has recently been shown to affect the biophysical properties of hair cells of the juvenile lateral line when used at 0.1% (Olt *et al*. 2014). We initially confirmed this result by testing a lower concentration of MS‐222 (0.017%), which is similar to that normally used to anaesthetize juvenile/adult fish for imaging and electrophysiological recordings (0.015–0.03%: Johnston *et al*. 2013; Barbosa *et al*. 2015; Xu *et al*. 2015). When MS‐222 was locally superfused onto the patched hair cells of 19–26 dpf decapitated zebrafish, it reversibly reduced the outward K^+^ current (Fig. 1 *A–D*). The size of the peak outward K^+^ currents (377 ± 46 pA, *n* = 7) was significantly reduced in the presence of 0.017% MS‐222 (245 ± 19 pA, *n* = 7, *P *< 0.02). Although MS‐222 does not affect larval hair cells (Olt *et al*. 2014), the above data confirm that it is not suitable for studying hair cell physiology in juvenile (young‐adult) zebrafish.

### The anaesthetic benzocaine does not affect hair cell physiology

In order to design a new experimental approach to investigate hair cell function in juvenile or adult zebrafish, we looked for a more suitable anaesthetic (Zahl *et al*. 2012; Collymore *et al*. 2014). Benzocaine was selected based on its analgesic properties and its safety (Neiffer & Stamper, 2009; Zahl *et al*. 2012). We tested a range of concentrations from 10 to 100 mg l^–1^ and found that 50 mg l^–1^ of benzocaine was sufficient to cause deep anaesthesia in juvenile zebrafish. Table 1 shows the different stages of anaesthesia (Zahl *et al*. 2012) reached by juvenile fish (42–47 dpf) using 50 mg l^–1^ or 100 mg l^–1^ benzocaine and MS‐222 at 0.017%, which were found to be comparable between the two anaesthetics.

**Figure 2:**
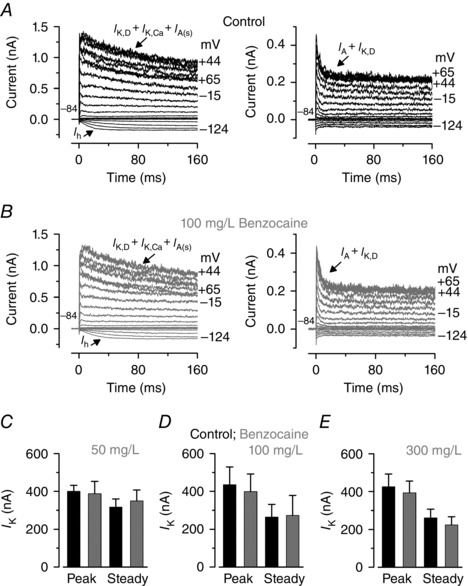
**Benzocaine did not block the K^+^ currents in hair cells from young‐adult zebrafish** *A* and *B*, characteristic K^+^ current recordings from lateral line hair cells positioned in the edge (left panels; 25 dpf) and centre (right panels; 25 dpf) of the neuromast before (top panels) and during (bottom panels) the local application of 100 mg l^–1^ benzocaine. Voltage protocol is as in Fig. 1. Note that hair cells recorded from the edge and centre of a neuromast show a different current profile, which has previously been characterized (Olt *et al*. 2014). All cells expressed a delayed rectifier K^+^ current (*I*_K,D_). However, most cells in the edge region expressed a Ca^2+^‐activated K^+^ current (*I*_K,Ca_), a small A‐type current (*I*_A(s)_) and an h‐type current (*I*_h_), whilst those in the centre expressed a large *I*_A_. *C–E*, average peak and steady‐state current measured near 0 mV before (black bars) and during the superfusion of different concentrations of benzocaine (grey bars) from 24–26 dpf zebrafish: *C*: control: peak 400 ± 32 pA, steady 317 ± 43 pA, *n* = 2; benzocaine (50 mg l^–1^): peak 388 ± 65 pA, steady 349 ± 58 pA, *n* = 2; *D*: control: peak 435 ± 94 pA, steady 265 ± 66 pA, *n* = 5; benzocaine (100 mg l^–1^): peak 399 ± 93 pA, steady 273 ± 105 pA, *n* = 5; *E*: control: peak 426 ± 67 pA, steady 261 ± 45 pA, *n* = 7; benzocaine (300 mg l^–1^): peak 393 ± 62 pA, steady 224 ± 43 pA, *n* = 7.

We then tested whether the biophysical properties of lateral line hair cells (24–26 dpf zebrafish) were affected by benzocaine. For these experiments, zebrafish were anaesthetized with MS‐222, decapitated and immediately washed from the anaesthetic with normal extracellular solution, an experimental condition that does not affect hair cell responses since the effects of MS‐222 are fully reversible (Olt *et al*. 2014). Hair cell physiology was investigated using three different concentrations of benzocaine, which was locally superfused onto cells during voltage clamp recordings. These were 50 mg l^–1^, which was the ideal concentration to anaesthetize zebrafish, 100 mg l^–1^ and 300 mg l^–1^ to test any possible dose‐dependent effect at higher concentrations. Examples of K^+^ currents recorded from hair cells of a 25 dpf zebrafish before and during the superfusion of 100 mg l^–1^ benzocaine are shown in Fig. 2 *A* and *B*, respectively. The two different current profiles depicted in Fig. 2 *A* (left and right) are due to the different positions of the hair cells within the neuromast (edge and centre, respectively; Olt *et al*. 2014). The average peak and steady‐state values of the K^+^ currents measured near 0 mV before and during the superfusion of the three different concentrations of benzocaine are shown in Fig. 2 *C–E*. The outward K^+^ current was not significantly affected by benzocaine, indicating that this anaesthetic does not block K^+^ channels in hair cells of young‐adult zebrafish even at a concentration six times higher than that needed to anaesthetize the fish (50 mg l^–1^).

**Figure 3:**
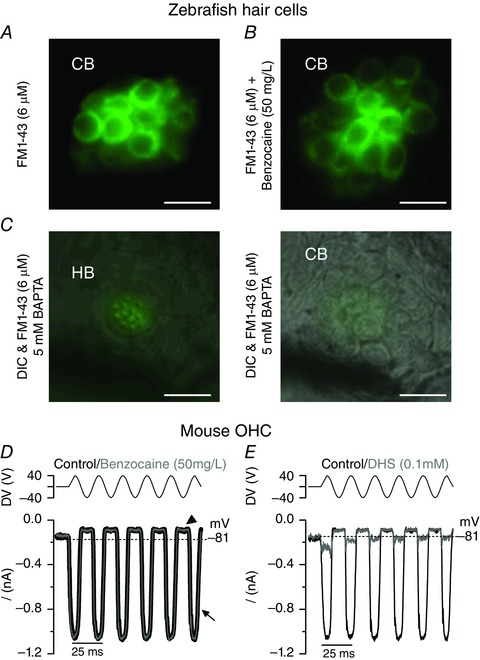
**Benzocaine does not affect the MET current in hair cells** *A* and *B*, fluorescence images from 14 dpf zebrafish neuromasts (primI) showing that hair cells take up FM1‐43 when applied alone (*A*) or together with 50 mg l^–1^ benzocaine (*B*). CB indicates the cell body of the hair cells within the neuromast. *C*, fluorescence images with the DIC image superimposed from 5 dpf zebrafish neuromasts treated for 5 min with 5 mm BAPTA before the application of FM1‐43. Note that FM1‐43 labelled the stereociliary hair bundle, most likely due to the partitioning of the dye in to the outer leaflet of the hair bundle plasma membrane (HC: left panel), but not the cell body (CB: right panel) of hair cells. Scale bars in *A–D*: 10 μm. *D* and *E*, mechanoelectrical transducer (MET) currents recorded from a P6 mouse OHC before (black trace) and during (grey trace) the superfusion of 50 mg l^–1^ benzocaine (*D*) and before (black trace) and during (grey trace) the superfusion of 100 μm dihydrostreptomycin (DHS) (*E*). MET currents were obtained by applying saturating sinusoidal force stimuli of 50 Hz to the hair bundles at −81 mV. The driver voltage (DV) signal of ±40 V to the fluid jet is shown above the traces (positive deflections of the DV are excitatory). The arrow indicates the inward MET current and the arrowhead the closure of the MET currents (i.e. resting MET current) elicited during inhibitory bundle displacements. Dashed lines indicate the holding current, which is the current at the holding membrane potential. [Colour figure can be viewed at wileyonlinelibrary.com]

The possible effect of benzocaine on the mechanoelectrical transducer (MET) current, which is crucial for sensory transduction in hair cells, was investigated using FM1‐43 (see Methods). FM1‐43 is a styryl dye known to permeate the open MET channel and has been previously used to assess the presence of a normal resting MET current (Gale *et al*. 2001; Kindt *et al*. 2012; Furness *et al*. 2013; Johnson, 2015). The use of FM1‐43 was chosen as a monitor of MET channel function in hair cells of the lateral line, since it is difficult to reliably record MET currents from individual cells (personal observations; Ricci *et al*. 2013). We found that FM1‐43 was able to enter and label hair cells of 14 dpf decapitated zebrafish when superfused alone (Fig. 3 *A*) or in the presence of 50 mg l^–1^ benzocaine (Fig. 3 *B*), indicating that the anaesthetic is unlikely to affect the MET current. Similar results were obtained in two additional zebrafish for each experimental condition. The selective uptake of FM1‐43 into the hair cells was prevented by perfusing the zebrafish with 5 mm BAPTA prior the application of the dye (Fig. 3 *C*, right panel). BAPTA is known to break the tip links and as such removes the resting open probability of the MET channel (see Methods). In order to provide more direct evidence that benzocaine does not block the MET channel, we performed MET current recordings from mouse OHCs, since mechanoelectrical transduction is highly conserved between zebrafish and rodents (Nicolson, 2005; Maeda *et al*. 2014). MET currents were recorded from P6 OHCs (Fig. 3 *D* and *E*) by displacing their hair bundles in the excitatory and inhibitory direction using a piezo‐driven fluid jet (Corns *et al*. 2016). At the membrane potentials of −81 mV, the displacement of the hair bundle in the excitatory direction (i.e. towards the taller stereocilia) elicited a large inward MET current (Fig. 3 *D*, arrow). The resting current flowing through open MET channels in the absence of mechanical stimulation was reduced when bundles were moved in the inhibitory direction (i.e. away from the taller stereocilia) (Fig. 3 *D*, arrowhead). We found that the maximal MET current was similar before (−880 ± 66 pA, *n* = 4) and during (−873 ± 68 pA, *n* = 4) the superfusion of 50 mg l^–1^ benzocaine. As a positive control, we superfused the same OHCs with 100 μm dihydrostreptomycin (DHS), a known blocker of the mechanotransducer channel (Marcotti *et al*. 2005), and found that the MET current was almost completely abolished (−102 ± 6 pA, *n* = 4; Fig. 3 *E*). These data obtained in mouse OHCs further support the fact that benzocaine, at the concentration used for *in vivo* work (50 mg l^–1^ = 0.3 mm) does not interfere with the MET current.

**Figure 4:**
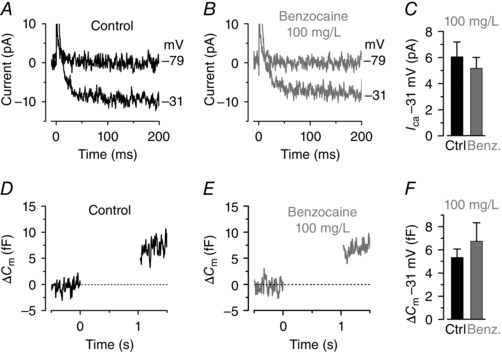
**Ca^2+^ currents and neurotransmitter release in lateral line hair cells is not affected by benzocaine** *A* and *B*, Ca^2+^ current (*I*_Ca_) recorded from hair cells of a 35 dpf zebrafish lateral line before (*A*) and during (*B*) the local superfusion of 100 mg l^–1^ benzocaine. Currents were elicited by a depolarizing voltage step to –31 mV (200 ms in duration) from the holding potential of –79 mV. *C*, average *I*_Ca_ from seven hair cells from 27–35 dpf decapitated zebrafish before (black) and during (grey) benzocaine. Control *I*_Ca_: 6.1 ± 1.1 pA, *n* = 7; *I*_Ca_ in benzocaine: 5.2 ± 0.8 pA, *n* = 7. *D* and *E*, changes in membrane capacitance (Δ*C*_m_) recorded from a hair cell of 35 dpf zebrafish. Recordings were obtained in response to 1 s voltage steps from the holding potential of −79 mV to near the peak of *I*_Ca_ (−31 mV). *F*, average Δ*C*_m_ recorded from four hair cells of 27–31 dpf zebrafish before (black) and during (grey) benzocaine. Control Δ*C*_m_: 5.3 ± 0.7 fF, *n* = 4; Δ*C*_m_ in benzocaine: 6.7 ± 1.6 pA, *n* = 4.

We then investigated whether benzocaine affected the synaptic activity of zebrafish hair cells by recording Ca^2+^ currents (*I*_Ca_) and induced exocytosis in 27–35 dpf zebrafish. We isolated *I*_Ca_ from the total membrane current in hair cells by blocking the K^+^ currents with 4‐AP and TEA in the caesium‐based intracellular solution (see Methods and Olt *et al*. 2014). *I*_Ca_ was recorded before and during the local application of 100 mg l^–1^ benzocaine (Fig. 4 *A* and *B*, respectively) using a 200 ms depolarizing voltage step to –31 mV from the holding potential of –79 mV, which elicited the maximal current (Olt *et al*. 2014). We found that the size of *I*_Ca_ was not significantly affected by benzocaine (Fig. 4 *A–C*). Calcium‐induced exocytosis was estimated by measuring increases in cell membrane capacitance (Δ*C*_m_) (Moser & Beutner, 2000; Olt *et al*. 2014) following 1 s depolarizing voltage steps to near –30 mV. We found that superfusion of 100 mg l^–1^ benzocaine (Fig. 4 *E* and *F*) did not significantly reduce Δ*C*_m_ compared to control recordings (Fig. 4 *D* and *F*). *I*_Ca_ and Δ*C*_m_ recordings were performed using 2.8 mm extracellular Ca^2+^ and at the physiological temperature for the zebrafish (28.5°C).

**Figure 5:**
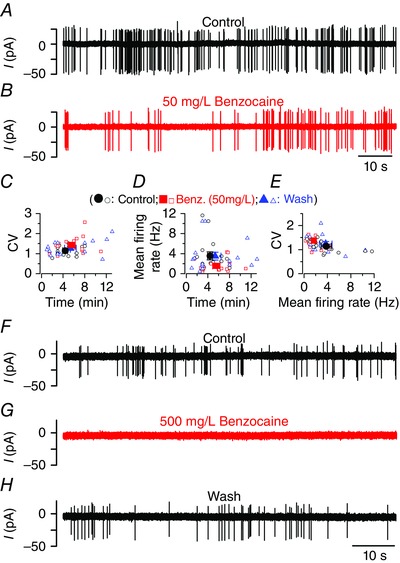
**Benzocaine affects the afferent activity in the zebrafish lateral line** *A* and *B*, spontaneous firing recorded with loose‐patch voltage clamp from the posterior lateral line ganglion (PLLg) of a 5 dpf zebrafish in the presence of normal extracellular solution (*A*) and during the application of 50 mg l^–1^ benzocaine (*B*). *C* and *D*, coefficient of variation (*C*, CV) and mean firing rate (*D*) as a function of recordings time. *E*, CV from each recording against the respective firing rate (single recording: open symbols; averages: filled symbols). The total duration of the recoding time and spike number for each experimental condition was: control 70.4 min and 14604 spikes, 50 mg l^–1^ benzocaine 89 min and 8753 spikes, wash 85.4 min and 17767 spikes. *F*–*H*, spontaneous firing from a PPLg of a 4.5 dpf zebrafish in normal extracellular solution (*F*), during the application of 500 mg l^–1^ benzocaine (*G*) and the final washout (*H*). [Colour figure can be viewed at wileyonlinelibrary.com]

After confirming that synaptic transmission at hair cell ribbon synapses remains unaltered in the presence of benzocaine, we tested whether the anaesthetic had any influence on the afferent neuron activity (Fig. 5). Spontaneous action potential activity was recorded from the cell body of the posterior lateral line ganglion (PLLg: Trapani & Nicolson, 2010; Trapani & Nicolson, 2011). For these experiments, larval zebrafish (4–5.2 dpf) were used because recordings from older fish require decapitation or the use of an anaesthetic throughout the experiment, which will either destroy the PLLg or alter the baseline firing activity, respectively. Loose‐patch electrophysiological recordings were, in 6 out of 16 cases, obtained with the sequential superfusion of the following solutions: normal extracellular solution (base‐line activity); 50 ml l^–1^ benzocaine (corresponding to 0.3 mm), normal extracellular solution, 500 ml l^–1^ benzocaine (3 mm) and a final wash. In the additional 10 recordings, only 50 mg l^–1^ benzocaine was tested. Figure 5 *A–E* shows that in the presence of 50 ml l^–1^ benzocaine the spike frequency of afferent neurons was highly reduced (1.56 ± 0.31 Hz, *n* = 16) compared to the control (3.57 ± 0.81 Hz, *n* = 16, *P *< 0.001 from repeated measures ANOVA followed by Bonferroni *post hoc* test: overall *P < *0.0001) and washout (3.51 ± 0.78 Hz, *n* = 16, *P *< 0.01). The spike frequency of the control and washout was not significantly different. The coefficient of variation, which provides a quantitative measure of regularity in spontaneous spike firing, was also significantly increased (overall repeated measures ANOVA: *P < *0.02) in the presence of benzocaine (1.43 ± 0.10, *n* = 16, 4–5 dpf) compared to the control conditions (control: 1.15 ± 0.06, *n* = 16; washout: 1.27 ± 0.09, *n* = 16). It is worth noting that both the firing frequency and CV in control conditions were found to be quite variable at these early stages of development (4–5 dpf: frequency 0.5–12 Hz; CV: 0.8–1.5), which supports the idea that, in addition to hair cells (Olt *et al*. 2014), also the afferent fibres contacting hair cells are still largely developing at these early stages (Haehnel *et al*. 2012; Liao & Haehnel, 2012). We conclude that 50 mg l^–1^ benzocaine affects the frequency and CV of the firing activity in the PLLg. In 500 ml l^–1^ benzocaine, the firing activity observed in control recordings (Fig. 5 *F*) was almost completely abolished within 59 ± 19 s (*n* = 6; Fig. 5 *G*). The effect of benzocaine was fully reversed in 111 ± 23 s (*n* = 6; Fig. 5 *H*).

### *In vivo* recordings from hair cells of young‐adult zebrafish

**Figure 6:**
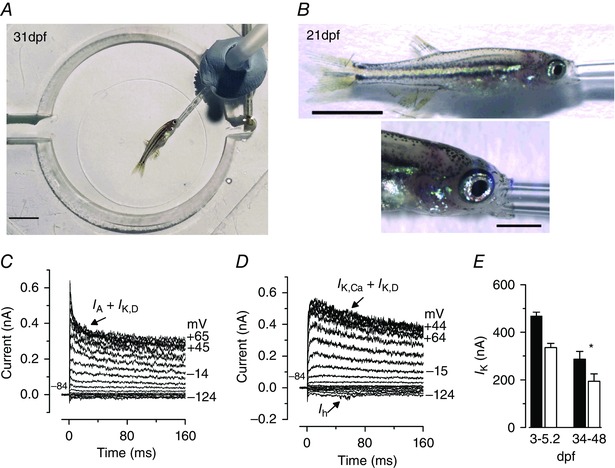
***In vivo* recordings from lateral line hair cells of young adult zebrafish** *A*, image showing a 31 dpf intubated zebrafish under anaesthesia (using 50 mg l^–1^ benzocaine) at the bottom of the microscope recording chamber. Scale bar: 0.4 mm. *B*, images showing the cannula inserted in the mouth of a 21 dpf zebrafish. Scale bar: 1 mm (top) and 0.5 mm (bottom). The cannula delivered extracellular solution that was not oxygenated. *C* and *D*, example of K^+^ currents recorded from hair cells of 34 dpf (*A*) and 48 dpf (*B*) zebrafish elicited using the same voltage protocol as in Fig. 1. *E*, comparison of the average size of the peak (black columns) and steady‐state (white columns) outward K^+^ current at 0 mV recorded *in vivo* from larval (from Olt *et al*. 2014) and juvenile zebrafish hair cells. Larval (3–5.2 dpf) hair cells: peak 468 ± 16 pA, steady 335 ± 18 pA, *n* = 41; juvenile (34–38) hair cells: peak 287 ± 32 pA, steady 194 ± 31 pA, *n* = 8). [Colour figure can be viewed at wileyonlinelibrary.com]

At larval stages, zebrafish maintain their blood oxygenation level via percutaneous breathing. However, respiration and blood oxygenation in zebrafish older than 21 dpf requires gill movement. Recent studies have shown that in adult zebrafish, which are normally > 2–3 cm long (Parichy *et al*. 2009), respiration can be maintained by intubating them with a standard cannula (≥ 1 mm in diameter; Johnston *et al*. 2013; Barbosa *et al*. 2015; Xu *et al*. 2015). One of the main benefits of using the zebrafish is that most of the transgenic lines are optically transparent, but this only applies to fish during the first few weeks post‐fertilization at juvenile stages, when the fish are much smaller (< 0.8 cm in length; Parichy *et al*. 2009; Olt *et al*. 2014). At this age, the lateral line contains functionally mature hair cells (Olt *et al*. 2014). In order to intubate these relatively small juvenile zebrafish (Fig. 6 *A* and *B*), the cannula was pulled to a diameter of 0.2–0.4 mm depending on the age of the zebrafish (fine bore polyethene tubing: 2 mm I.D.; 3 mm O.D.). After reaching full anaesthesia with benzocaine (stage V, see Table 1), the cannula was inserted carefully into the mouth of the zebrafish and the extracellular solution delivered with a flow rate of ∼1 ml min^–1^. Under these *in vivo* recording conditions, hair cells from juvenile fish expressed K^+^ currents (Fig. 6 *C* and *D*) that were qualitatively similar, but significantly smaller, than those recorded *in vivo* from hair cells of the larval lateral line zebrafish (Fig. 6 *E*). This finding, together with the fact that the proportion in the different current profile changes with the maturation of the lateral line (Olt *et al*. 2014), further supports the evidence that larval neuromasts are still undergoing developmental changes at larval stages.

## Discussion

We describe an experimental method to record from functionally mature hair cells of live juvenile zebrafish. We found that benzocaine at 50 mg l^–1^ is an ideal anaesthetic in terms of its effectiveness as an analgesic and its safety for both humans and fish. This approach, combined with intubation of juvenile zebrafish to maintain gill function, allows us to perform *in vivo* electrophysiological studies from hair cells of the functionally mature lateral line at an age when the zebrafish are still optically transparent. Thus the zebrafish provides an excellent model to investigate the molecular mechanisms underlying sensory transduction and signal encoding in mature hair cells *in vivo*, which is extremely difficult to do in mammals.

### The potential for *in vivo* recordings from a functionally mature system

The zebrafish (*Danio rerio*) is becoming a powerful model not only for genetic analysis (Phillips & Westerfield, 2014) but also for functional *in vivo* studies involving behaviour (Kalueff *et al*. 2014; Stewart *et al*. 2014; Tabor *et al*. 2014), optical imaging and electrophysiology (motoneurons: Ampatzis *et al*. 2013; Fidelin & Wyart, 2014; retina horizontal and ganglion cells: Sun *et al*. 2012; Johnston *et al*. 2014; hair cells: Olt *et al*. 2014). However, the full potential of the zebrafish for functional *in vivo* studies is limited by the fact that most of the work is carried out at larval stages (e.g. Liao & Haehnel, 2012; Ampatzis *et al*. 2013). In the zebrafish lateral line, the majority of hair cells only reach functional maturity in terms of basolateral ion channel profile and synaptic activity at juvenile stages (Olt *et al*. 2014). Similarly, the afferent fibres contacting hair cells undergo extensive growth and reorganization after larval stage, which is essential for the fine‐tuning and increased sensitivity to movement of adult zebrafish (Haehnel *et al*. 2012; Liao & Haehnel, 2012).

The choice of anaesthetic to be used for *in vivo* experiments is critical since it could alter the normal physiology of sensory cells and neurons. We have shown that the anaesthetic MS‐222, the preferred choice for both larval and adult zebrafish based on its efficacy (e.g. Collymore *et al*. 2014), blocks the K^+^ currents expressed in juvenile zebrafish hair cells (Fig. 1) and as such cannot be used to study the *in vivo* physiology of functionally mature cells. Benzocaine is an ideal alternative to MS‐222 since it did not affect the biophysical properties of juvenile hair cells (Figs 2, 3, 4). However, both MS‐222 and benzocaine induce anaesthesia by blocking voltage‐sensitive Na^+^ channels (Frazier & Narahashi, 1975; Neumcke *et al*. 1981), which is likely to impact directly on the firing activity of neurons. At concentrations used for *in vivo* experiments (MS‐222: > 0.01%; benzocaine: 50 mg l^–1^), both anaesthetics significantly decrease the spontaneous firing rate and affect the regularity (CV) of the afferent firing activity in the lateral line of the Toadfish (Palmer & Mensinger, 2004) and zebrafish (Fig. 5). While the effect of benzocaine on the firing activity was completely reversible within a few minutes, that of MS‐222 require a longer recovery (about 90 min: Palmer & Mensinger, 2004). Therefore, the experimental method described in this study allows us to perform electrophysiological recordings from the functionally mature hair cells of the zebrafish lateral line, which can be done in combination with imaging techniques using genetically targeted fluorescent proteins (Dreosti & Lagnado, 2011). This is because most zebrafish are optically transparent or have low opacity during the first few weeks post‐fertilization, making structure inside the fish accessible for imaging. Because of the mechanism of action of anaesthetics, both benzocaine and MS‐222 are not suitable for investigating neural activity. This includes looking at efferent feedback modulation of hair cell activity (Toro *et al*. 2015) and how activity from different hair cells within one or more neuromasts is integrated higher up in the sensory pathway, in the mature lateral line *in vivo*.

### Is the zebrafish a useful alternative to mammals for investigating hair cell function?

The lateral line senses vibration and water movement around the body, which is equivalent to the function of vestibular organs in lower vertebrates and mammals. From the biophysical point of view, the hair cells of the lateral line resemble those found in the utricles of the mouse (Rüsch *et al*. 1998) and pigeon (Weng & Correia, 1999) as well as those in the auditory organs of other lower vertebrates (e.g. goldfish sacculus: Sugihara & Furukawa, 1989, 1996). The current profile of mature lateral line hair cells (Figs 2, 4 and 6; Olt *et al*. 2014) largely differs from that found in mouse cochlea hair cells (Marcotti & Kros, 1999; Marcotti *et al*. 2003, 2004), suggesting that the two systems are unlikely to be fully comparable. However, mechanoelectrical transduction is highly conserved in auditory and vestibular systems of lower vertebrates and mammals (Nicolson, 2005; Fettiplace & Kim, 2014; Maeda *et al*. 2014), as is much of the basic architecture of ribbon synapses (Nicolson, 2015). The ability to combine genetic manipulation with electrophysiology and imaging from functionally mature hair cells of living zebrafish will provide a unique experimental platform towards an understanding of the molecular mechanisms underlying sensory transduction and signal encoding.

## Additional information

### Competing interests

None declared.

### Author contributions

Conception and design of the experiments: J.O., W.M. Collection and analysis of data: JO, CEA, WM. Writing the paper: JO, WM. All authors have approved the final version of the manuscript and agree to be accountable for all aspects of the work. All persons designated as authors qualify for authorship, and all those who qualify for authorship are listed.

### Funding

This work was supported by a Wellcome Trust grant (102892) to W.M. and a Boerhinger Ingelheim travel award to J.O. A PhD studentship to J.O. was supported by the University of Sheffield.
